# Integrated Immune Escape in Cervical Cancer: HLA-I Dysfunction and Immune Checkpoint Signaling

**DOI:** 10.3390/ijms27188009

**Published:** 2026-09-09

**Authors:** Angel Yordanov, Vasilena Dimitrova Dimitrova

**Affiliations:** Department of Gynaecological Oncology, Medical University Pleven, 5800 Pleven, Bulgaria

**Keywords:** cervical cancer, human papillomavirus, HLA class I, CD8^+^ T lymphocytes, FOXP3, PD-1, PD-L1, CTLA-4, immune escape, immunotherapy

## Abstract

Persistent infection with high-risk human papillomavirus (hrHPV) is the principal cause of cervical cancer and initiates a complex process of immune evasion that enables malignant progression despite continuous expression of the viral oncoproteins E6 and E7. Effective antitumor immunity depends on coordinated antigen presentation through human leukocyte antigen class I (HLA-I) molecules, activation of CD8^+^ cytotoxic T lymphocytes, and balanced regulation of immune responses. During cervical carcinogenesis, these mechanisms are progressively disrupted through HLA-I downregulation, CD8^+^ T-cell exhaustion, expansion of FOXP3^+^ regulatory T cells, and activation of the PD-1/PD-L1 and CTLA-4 immune checkpoint pathways, ultimately establishing an immunosuppressive tumor microenvironment that promotes immune escape. This narrative review integrates current evidence on HLA-I-mediated antigen presentation, CD8^+^ cytotoxic T-cell function, FOXP3^+^ regulatory T cells, and immune checkpoint signaling into a unified model of immune escape during cervical carcinogenesis. In addition, it discusses the clinical implications of these interconnected mechanisms, including immune checkpoint inhibition, emerging therapeutic strategies, integrated immune profiling, and future directions in personalized immunotherapy. A comprehensive understanding of the interactions between antigen presentation, immune-cell function, and immune checkpoint regulation provides the biological foundation for current and future immunotherapeutic approaches. Integrating molecular, cellular, and spatial immune characteristics may improve patient stratification, optimize treatment selection, and facilitate the implementation of precision immuno-oncology in cervical cancer.

## 1. Introduction

Cervical cancer remains a major global health problem, with approximately 660,000 new cases and more than 350,000 deaths estimated worldwide in 2022; close to 90% of this burden occurs in low- and middle-income countries [1]. HPV acquisition is common, but most infections are transient. Persistent infection with a high-risk genotype is the relevant carcinogenic event, and HPV16 and HPV18 together account for approximately 70% of cervical cancers [2,3,4,5,6,7,8].

Persistent infection with a high-risk HPV genotype, rather than HPV exposure itself, is the necessary initiating condition for cervical carcinogenesis [6,7,8]. Harald zur Hausen’s work establishing this causal relationship changed the field and provided the rationale for prophylactic vaccination [9]. Yet, persistence is uncommon: most infections are cleared by host immunity, whereas a minority evade control and accumulate the molecular changes that permit malignant transformation [6,7,8].

This difference between frequent infection and comparatively rare cancer places immune surveillance at the center of disease progression. Viral oncogene activity is indispensable, but it is the continuing interaction between HPV-infected epithelial cells and the host immune system that determines whether infection is cleared, contained, or allowed to progress.

Two components of this interaction are especially important: presentation of viral antigens through human leukocyte antigen class I (HLA-I) molecules and regulation of T-cell activity by immune checkpoints. These are not parallel, independent processes. HLA-I determines whether a tumor cell can be recognized, whereas PD-1/PD-L1, CTLA-4, FOXP3^+^ regulatory T cells, and the functional state of CD8^+^ T cells determine whether recognition produces an effective response [10,11,12,13].

The clinical activity of immune checkpoint inhibitors in recurrent and metastatic cervical cancer confirms the therapeutic relevance of this biology, but the marked variability in response also exposes its limits. Releasing an inhibitory checkpoint cannot compensate for every defect in antigen presentation, T-cell trafficking, or local immune suppression. This makes the tumor immune microenvironment a problem of interacting resistance mechanisms, not a single target.

Most of these pathways have been studied extensively, although often in isolation. Several unresolved observations show why this separation is insufficient: PD-L1 may indicate either IFN-γ-driven immune engagement or adaptive suppression; abundant CD8^+^ cells may be excluded from tumor nests or functionally exhausted; and checkpoint release may have little effect when the relevant peptide–HLA-I complex is absent. These context-dependent meanings help explain why apparently inflamed tumors can fail to respond and why individual biomarkers have inconsistent prognostic or predictive performance [10,11,12,13].

Previous reviews have provided broad accounts of recent advances in cervical cancer, HPV E6/E7-mediated innate immune evasion, or the suppressive tumor microenvironment [11,12,13]. The specific contribution of the present review is to organize these observations as a conditional sequence: viral antigens must first be presented through HLA-I, CD8^+^ T cells must reach and remain functional within tumor tissue, and checkpoint and regulatory signals must not dominate the response. This hierarchy distinguishes failure of tumor recognition from inhibition after recognition and provides a mechanistic basis for interpreting resistance to single biomarkers and single-pathway therapy.

This narrative review therefore examines HLA-I-mediated antigen presentation, CD8^+^ T-cell dysfunction, FOXP3^+^ regulatory T cells, and PD-1/PD-L1 and CTLA-4 signaling as parts of one immune-escape system. The purpose is not only to summarize their established biology, but also to identify conflicting observations, translational bottlenecks, and the conditions under which integrated immune profiling might improve prognosis or treatment selection.

These axes were selected because they form a mechanistically connected sequence from tumor recognition to effector inhibition. The review is therefore not intended as an exhaustive atlas of every immune population or inhibitory receptor in cervical cancer. Natural killer (NK) cells, myeloid-derived suppressor cells (MDSCs), tumor-associated macrophages (TAMs), and additional checkpoints such as LAG-3, TIM-3, and TIGIT are considered where they modify this central sequence but are not reviewed comprehensively.

## 2. Literature Identification and Scope

In preparing this narrative review, we used PubMed/MEDLINE and Google Scholar to identify relevant English-language publications published between January 2000 and 31 May 2026. Separate searches were conducted for the main topics covered in the review. The terms ‘cervical cancer’, ‘cervical carcinoma’, ‘human papillomavirus’, and ‘HPV’ were combined with terms such as ‘HLA class I’, ‘antigen presentation’, ‘immune escape’, ‘CD8 T cells’, ‘T-cell exhaustion’, ‘FOXP3’, ‘regulatory T cells’, ‘PD-1’, ‘PD-L1’, ‘CTLA-4’, ‘tumor immune microenvironment’, and ‘immunotherapy’. We also examined the reference lists of key articles to identify additional relevant studies.

We focused on publications that helped explain the relationships between HPV persistence, impaired antigen presentation, T-cell dysfunction, immune checkpoint signaling, and response to immunotherapy in cervical cancer. The literature included mechanistic and translational studies, observational investigations, major clinical trials, and recent reviews. Evidence from other HPV-associated cancers or experimental models was considered only when it provided information directly relevant to cervical cancer. As the purpose was to develop a focused narrative synthesis rather than an exhaustive systematic review, no registered protocol, PRISMA-based selection procedure, or formal risk-of-bias assessment was used.

## 3. HPV-Driven Carcinogenesis

Cervical carcinogenesis is initiated by persistent infection with a high-risk HPV genotype. HPV16 and HPV18 account for approximately 70% of cervical cancers, while HPV31, HPV33, HPV45, HPV52, HPV58, and other high-risk types contribute to most of the remaining cases [6,7,8]. The distinction between infection and persistence is important. HPV exposure is common and, in most women, transient. Progression becomes biologically relevant when the virus is not cleared and the infected epithelium remains exposed to sustained viral oncogene activity over time [6,7,8].

Integration of viral DNA into the host genome is an important, although not obligatory, step in this process. Disruption of the viral E2 gene may release E6 and E7 from transcriptional control and promote persistent oncogene expression [10]. The downstream effects of E6 and E7 are well established: E6 promotes p53 degradation and weakens apoptosis and DNA-damage responses, whereas E7 inactivates the retinoblastoma protein, releases E2F transcription factors, and drives cell-cycle progression and genomic instability [10,11]. These events explain much of the transforming activity of high-risk HPV, but they do not fully explain why cells that continue to express viral proteins are able to persist in an immunocompetent host.

An important feature of HPV-associated cervical carcinogenesis is that oncogenic transformation develops in parallel with progressive alteration of local immune control. High-risk HPV oncoproteins interfere with innate antiviral signaling, dendritic-cell maturation, and type I interferon responses [11,12]. The consequence is not simply weaker antiviral defense. Persistent infection develops within an epithelial environment in which the signals required to recruit, activate, and coordinate immune responses have already been modified. This helps explain why immune escape in cervical cancer begins before the establishment of an overtly invasive tumor.

The effect is also evident at the epithelial–immune interface. HPV16 E6/E7 expression reduces keratinocyte production of CCL20/MIP-3α and thereby limits CCR6-dependent recruitment of Langerhans-cell precursors [14]. This is particularly relevant in the cervix, where Langerhans cells are positioned within the epithelium to capture viral antigens and participate in the initiation of adaptive immunity. Reduced recruitment or impaired activation of these cells may therefore weaken the early transition from recognition of HPV-infected keratinocytes to effective T-cell priming.

Importantly, this defect appears to be at least partly reversible. In women with CIN2/3, HPV16-exposed Langerhans cells have shown reduced costimulatory-molecule expression, inflammatory cytokine production, and chemokine-directed migration. Activation with a stabilized TLR3 agonist restored these functions and enabled HPV16-specific CD8^+^ T-cell priming [15]. These findings suggest that HPV-associated immune dysfunction is not uniformly caused by irreversible loss of immune function. At least in premalignant lesions, part of the defect may reflect a suppressed or inadequately activated local immune state.

**Figure 1:**
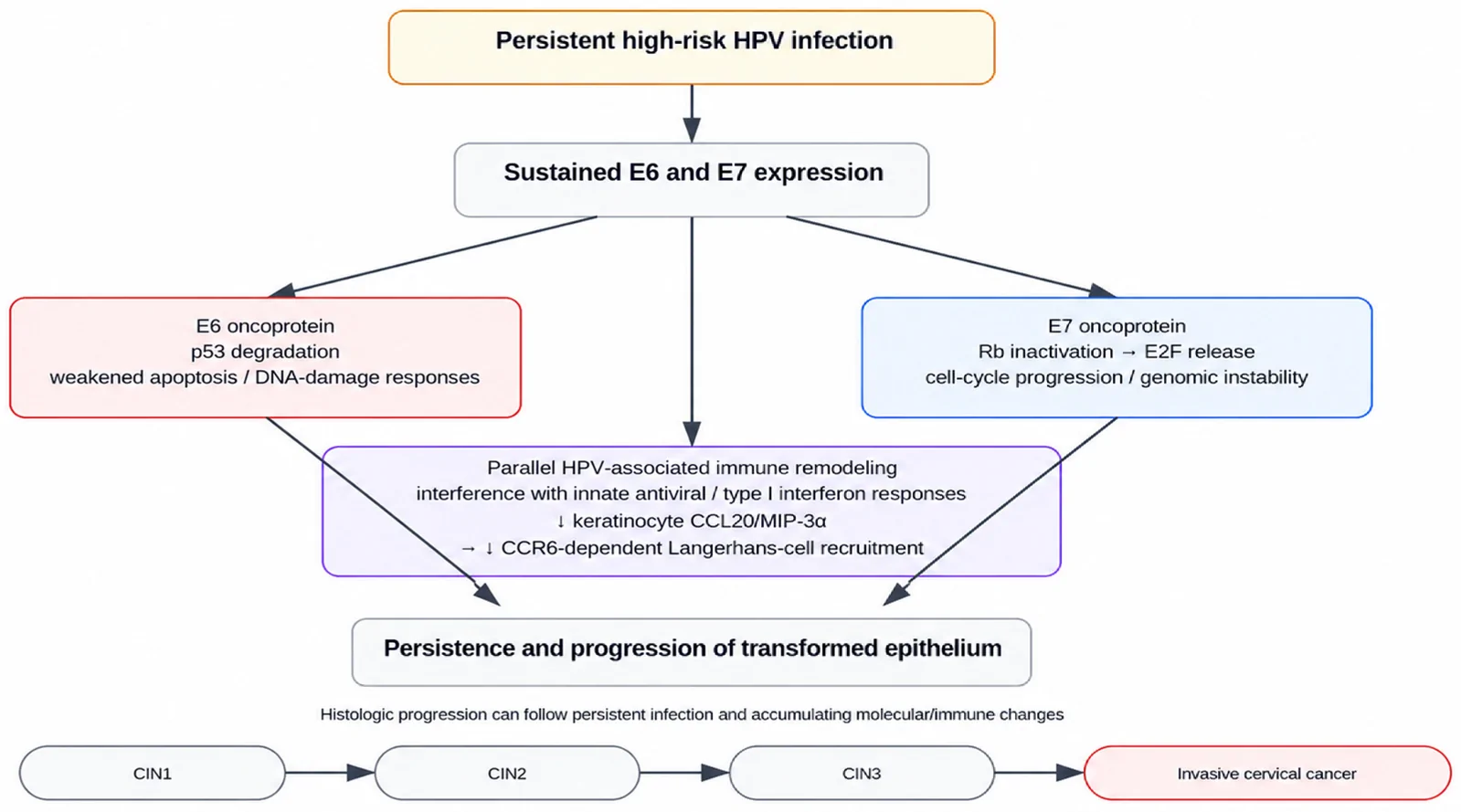
**HPV-driven cervical carcinogenesis and early immune remodeling**. Persistent high-risk HPV infection sustains E6 and E7 expression. E6 promotes p53 degradation and impairs apoptosis and DNA-damage responses, whereas E7 inactivates Rb, releases E2F transcription factors, and promotes cell-cycle progression and genomic instability. In parallel, HPV-associated epithelial immune remodeling alters innate antiviral signaling and reduces CCL20/MIP-3α-dependent recruitment of Langerhans-cell precursors, contributing to persistence and progression of transformed epithelium.

This early immune remodeling is relevant to the mechanisms discussed in the following sections. Reduced HLA-I-mediated antigen presentation, ineffective CD8^+^ T-cell responses, expansion of regulatory T-cell activity, and checkpoint signaling do not arise in isolation. They develop against a background of persistent viral antigen expression, altered epithelial signaling, and impaired communication between HPV-infected cells and the local immune system. Cervical cancer immune escape is therefore better understood as a progressive process in which defects in recognition, immune-cell recruitment, effector function, and inhibitory signaling accumulate and reinforce one another (Figure 1).

## 4. HLA-Mediated Antigen Presentation

Persistent expression of the HPV E6 and E7 oncoproteins should, in principle, provide a continuous source of tumor-associated viral antigens. Whether these antigens can be recognized by CD8^+^ T cells, however, depends on their effective presentation by human leukocyte antigen class I (HLA-I) molecules. This makes HLA-I expression a central point of vulnerability in HPV-associated cervical cancer: viral antigens may remain present while the malignant cell becomes progressively less visible to the immune system [13,16].

HLA-I molecules, including HLA-A, HLA-B, and HLA-C, present intracellularly derived peptides to CD8^+^ T cells. HPV-derived peptides generated from E6 and E7 are processed through the antigen-presentation machinery, including TAP1 and TAP2, before being loaded onto HLA-I molecules and transported to the cell surface [16,17]. In cervical cancer, the clinically relevant issue is therefore not the absence of recognizable viral proteins, but disruption of the machinery required to display them.

Several abnormalities can contribute to this loss of visibility. Cervical lesions may show reduced HLA-I heavy-chain expression, β2-microglobulin deficiency, impaired TAP activity, or defects in other components of the antigen-processing pathway [16,17,18]. These abnormalities are not equivalent. Some are potentially reversible and may reflect altered transcriptional or antigen-processing states, whereas structural loss of an HLA allele or β2-microglobulin is unlikely to be restored simply by enhancing T-cell activity [19]. This distinction is particularly important when considering immunotherapy, because checkpoint blockade can only reactivate T cells if the relevant tumor antigen is still being presented.

HLA-I loss in cervical cancer is also heterogeneous rather than uniform. Partial, allele-specific, and complete loss have all been reported, and the pattern observed in a primary cervical tumor may differ from that in corresponding lymph-node metastases [19,20]. Such variation suggests that impaired antigen presentation is shaped not only by viral biology but also by immune selection during tumor progression. A tumor clone that reduces presentation of a relevant HPV-derived peptide may gain a survival advantage under T-cell pressure, while other HLA molecules remain detectable. For this reason, a simple classification of tumors as “HLA-positive” or “HLA-negative” may obscure biologically important differences in the quality of antigen presentation.

This heterogeneity also complicates interpretation of immune-cell infiltration. The presence of activated or PD-1-positive CD8^+^ T cells does not necessarily indicate that they can recognize the malignant cells around them. A tumor may contain apparently engaged effector cells yet remain functionally inaccessible if the relevant peptide–HLA complex has been lost. HLA-I status may therefore help explain why some immune-infiltrated cervical tumors nevertheless fail to mount effective cytotoxic responses or respond incompletely to checkpoint inhibition [13,17,18,19,20]. At present, however, HLA-I assessment is not standardized, and the available clinical evidence remains largely retrospective. HLA-I abnormalities should therefore be considered biologically plausible resistance mechanisms rather than established predictive biomarkers.

Loss of classical HLA-I also changes the pressure exerted by other immune populations. Reduced HLA-I expression can favor natural killer (NK)-cell recognition through the “missing-self” mechanism, because inhibitory signaling through killer-cell immunoglobulin-like receptors is diminished [21]. This potential vulnerability does not necessarily translate into effective tumor clearance. Cervical tumors may retain or increase expression of non-classical HLA molecules such as HLA-E and HLA-G. HLA-E can inhibit NK cells and CD8^+^ T cells through CD94/NKG2A, whereas HLA-G can engage inhibitory receptors including ILT2 [20,22,23]. Thus, loss of classical HLA-I may remove one route of immune inhibition while simultaneously favoring alternative mechanisms of immune escape.

**Figure 2:**
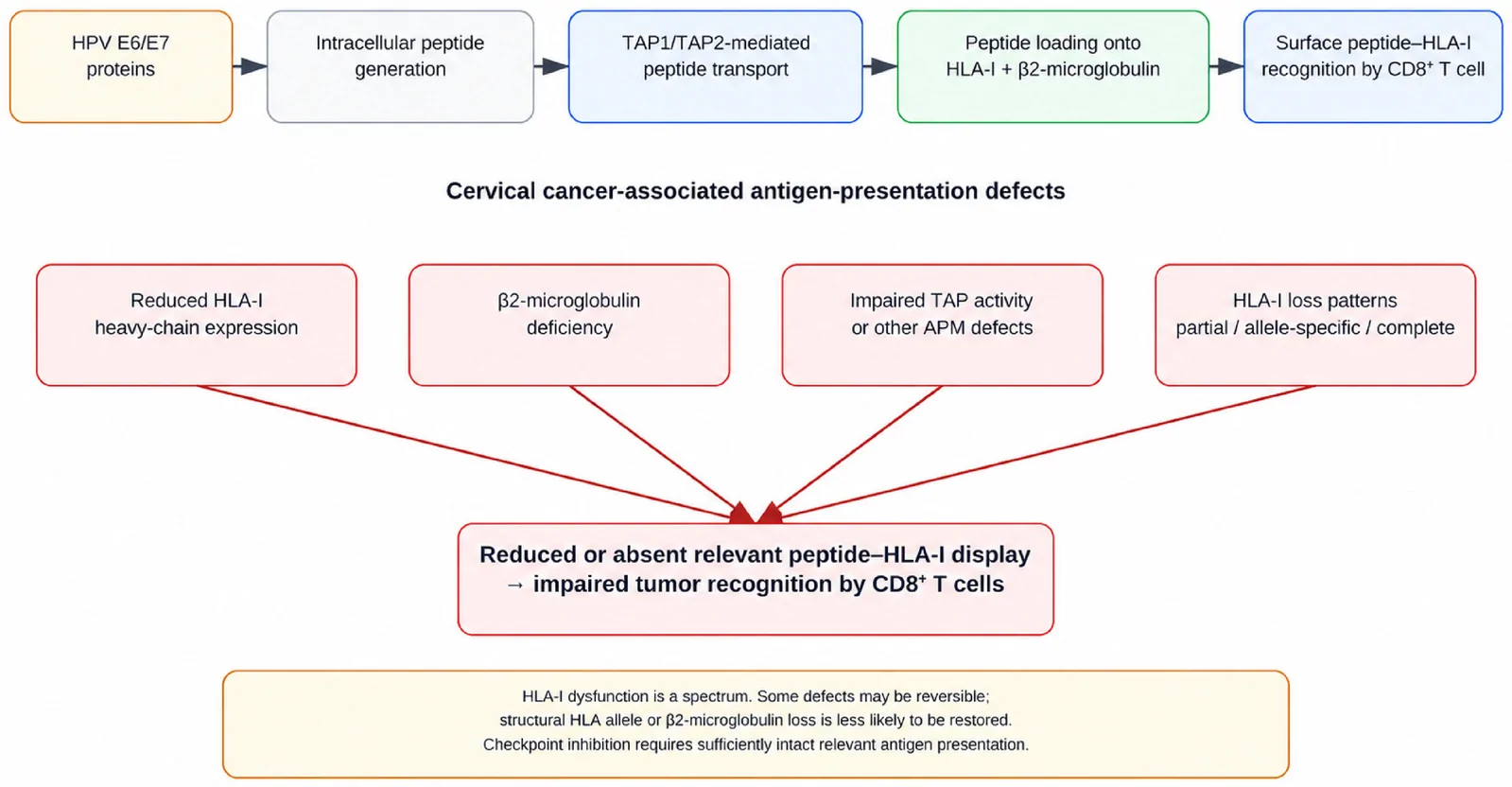
**HLA-I-mediated antigen presentation and recognition-deficient immune escape.** HPV-derived intracellular peptides are processed and transported through the antigen-presentation machinery before loading onto HLA-I molecules for recognition by CD8^+^ T cells. Reduced HLA-I heavy-chain expression, β2-microglobulin deficiency, impaired TAP activity, or partial, allele-specific, or complete HLA-I loss can reduce relevant peptide–HLA-I display and thereby impair tumor recognition.

As summarized in Figure 2, HLA-I dysfunction in cervical cancer is better viewed as a spectrum of antigen-presentation states than as a single molecular defect. The biological consequence depends on which component of the pathway is altered, whether the defect is reversible or structural, and how classical HLA-I loss is balanced by non-classical HLA expression and local immune-cell composition. This context is essential when interpreting subsequent CD8^+^ T-cell dysfunction and checkpoint activation, because inhibitory signaling becomes therapeutically relevant only when tumor recognition remains sufficiently intact.

## 5. CD8^+^ Cytotoxic T Cells

CD8^+^ T cells are the principal adaptive effector population capable of eliminating HPV-transformed cells once viral peptides are presented through HLA-I. In cervical cancer, however, their biological relevance cannot be judged simply by whether they are present in the tumor. The key questions are whether they can reach malignant cells, whether the relevant peptide–HLA-I complexes remain available for recognition, and whether the infiltrating T cells retain sufficient functional capacity to respond [11,24].

This distinction is important because CD8^+^ lymphocytes are not distributed uniformly within cervical tumors. Some are located within tumor nests, where direct contact with malignant cells is possible, whereas others remain confined to the surrounding stroma [13,24]. A tumor with abundant stromal CD8^+^ cells may therefore appear immunologically active while still maintaining an effective barrier between effector cells and their targets. In this setting, immune-cell density alone may overestimate the strength of the antitumor response.

Spatial access is only one part of the problem. Persistent exposure to HPV-derived antigens and prolonged inhibitory signaling can progressively reduce CD8^+^ T-cell function. Exhausted T cells show impaired proliferation, reduced cytokine production, and diminished cytotoxic activity, even when they remain detectable within the tissue [11,24,25]. Cervical tumors may consequently contain substantial numbers of CD8^+^ lymphocytes without generating an effective immune response against the malignant epithelium.

The interpretation of CD8^+^ infiltration therefore depends on the biological context in which these cells are found. High CD8^+^ density may be favorable when HLA-I-mediated recognition is preserved, the cells enter tumor nests, and their effector function remains intact. The same finding may have a very different meaning when HLA-I expression is reduced, when lymphocytes are restricted to the stroma, or when chronic checkpoint signaling has driven them toward a dysfunctional state. This helps explain why CD8^+^ cell counts alone have not provided a uniform prognostic or predictive signal across cervical cancer studies [13,24,25].

CD8^+^ T-cell dysfunction in cervical cancer is thus better understood as a problem of both location and state rather than cell number alone. Antigen presentation determines whether tumor cells can be recognized, spatial organization determines whether effector cells can physically reach their targets, and chronic inhibitory signaling determines whether recognition can still produce a functional response. These factors also provide the link between HLA-I abnormalities and the regulatory and checkpoint pathways discussed in the following sections.

## 6. FOXP3^+^ Regulatory T Cells

FOXP3^+^ regulatory T cells (Tregs) represent an important component of immune suppression in cervical cancer, but their biological significance cannot be inferred from cell number alone. Tregs suppress antitumor immunity through several complementary mechanisms, including secretion of IL-10 and TGF-β, consumption of IL-2, and CTLA-4-dependent inhibition of antigen-presenting-cell function [26,27]. In the cervical tumor microenvironment, these mechanisms act in the setting of persistent HPV antigen exposure and can restrict both the expansion and local activity of tumor-reactive T cells.

As with CD8^+^ lymphocytes, spatial distribution is important. FOXP3^+^ cells may be located within tumor nests or predominantly in the surrounding stroma, and these patterns are unlikely to be biologically equivalent [13,26,27]. Intratumoral Tregs are positioned to suppress effector cells in close proximity to malignant cells, whereas stromal Tregs may act earlier by limiting immune-cell activation and recruitment before cytotoxic lymphocytes reach the tumor compartment. This spatial distinction may help explain why associations between FOXP3^+^ cell density and clinical outcome have been inconsistent across cervical cancer studies.

**Figure 3:**
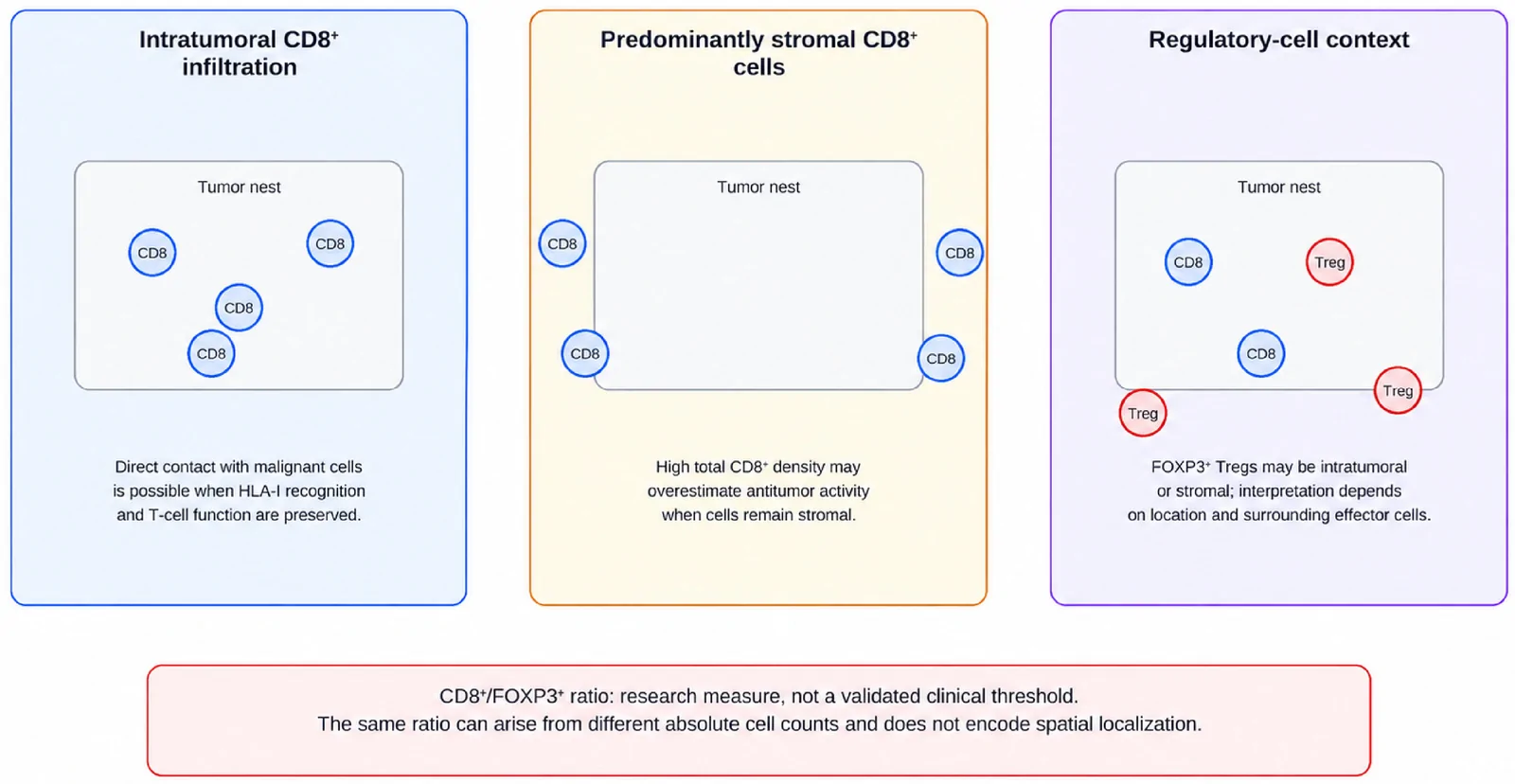
**Spatial interpretation of the CD8^+^–FOXP3^+^ immune balance in cervical cancer.** CD8^+^ T cells may infiltrate malignant nests or remain predominantly stromal, whereas FOXP3^+^ regulatory T cells may occupy either compartment. The biological meaning of the CD8^+^/FOXP3^+^ ratio therefore depends on absolute cell density and spatial localization; the schematic does not imply a validated clinical or prognostic threshold.

For this reason, the balance between cytotoxic and regulatory lymphocytes is often considered more informative than either population alone. The CD8^+^/FOXP3^+^ ratio provides a simple estimate of this balance and has shown prognostic associations in cervical cancer cohorts [24,26,27,28]. As illustrated in Figure 3, however, the biological meaning of this ratio depends not only on the relative abundance of CD8^+^ and FOXP3^+^ cells but also on their absolute density and spatial localization within tumor and stromal compartments. However, the same ratio can arise from very different absolute cell densities and does not indicate whether CD8^+^ cells have actually entered tumor nests. A high ratio in a sparsely infiltrated tumor is therefore not biologically equivalent to the same ratio in a densely inflamed tumor.

The apparent simplicity of the ratio also creates methodological limitations. Reported cut-offs are often derived retrospectively, ratios become unstable when FOXP3^+^ counts are very low, and results are sensitive to antibody selection, field selection, tumor-versus-stroma segmentation, and counting methodology [28,29]. Stage, histology, HPV-related factors, previous treatment, and the amount of tissue analyzed may further influence the observed association. The CD8^+^/FOXP3^+^ ratio should therefore be regarded as a biologically plausible research marker rather than a validated clinical biomarker.

Within the integrated model proposed here, Treg-mediated suppression is most relevant when tumor recognition and effector-cell access are at least partly preserved. Reducing regulatory suppression is unlikely to restore antitumor immunity if malignant cells have already escaped recognition through substantial HLA-I loss or if CD8^+^ T cells remain spatially excluded. FOXP3^+^ Tregs should therefore be interpreted as one layer of immune resistance whose impact depends on the upstream state of antigen presentation and the local relationship between regulatory and cytotoxic T cells.

## 7. PD-1/PD-L1 Pathway

The PD-1/PD-L1 pathway is particularly relevant in cervical cancer because it operates in the setting of persistent viral antigen exposure. HPV-derived antigens can maintain T-cell stimulation over prolonged periods, but sustained stimulation also favors the progressive engagement of inhibitory pathways. PD-1 expression on tumor-infiltrating T cells is therefore common in HPV-associated tumors and should not, on its own, be taken as evidence of irreversible exhaustion [11,25,30].

PD-L1 is similarly difficult to interpret in isolation. It can be expressed by malignant cells as well as by macrophages, dendritic cells, and other immune cells within the cervical tumor microenvironment [11,30]. Its expression may also be induced by IFN-γ released from activated T cells. In that setting, PD-L1 is not simply a constitutive mechanism of tumor escape but part of an adaptive response to immune pressure [11,12,30]. A PD-L1-positive cervical tumor may therefore contain an active antitumor response while simultaneously using that response to reinforce local immune suppression.

**Figure 4:**
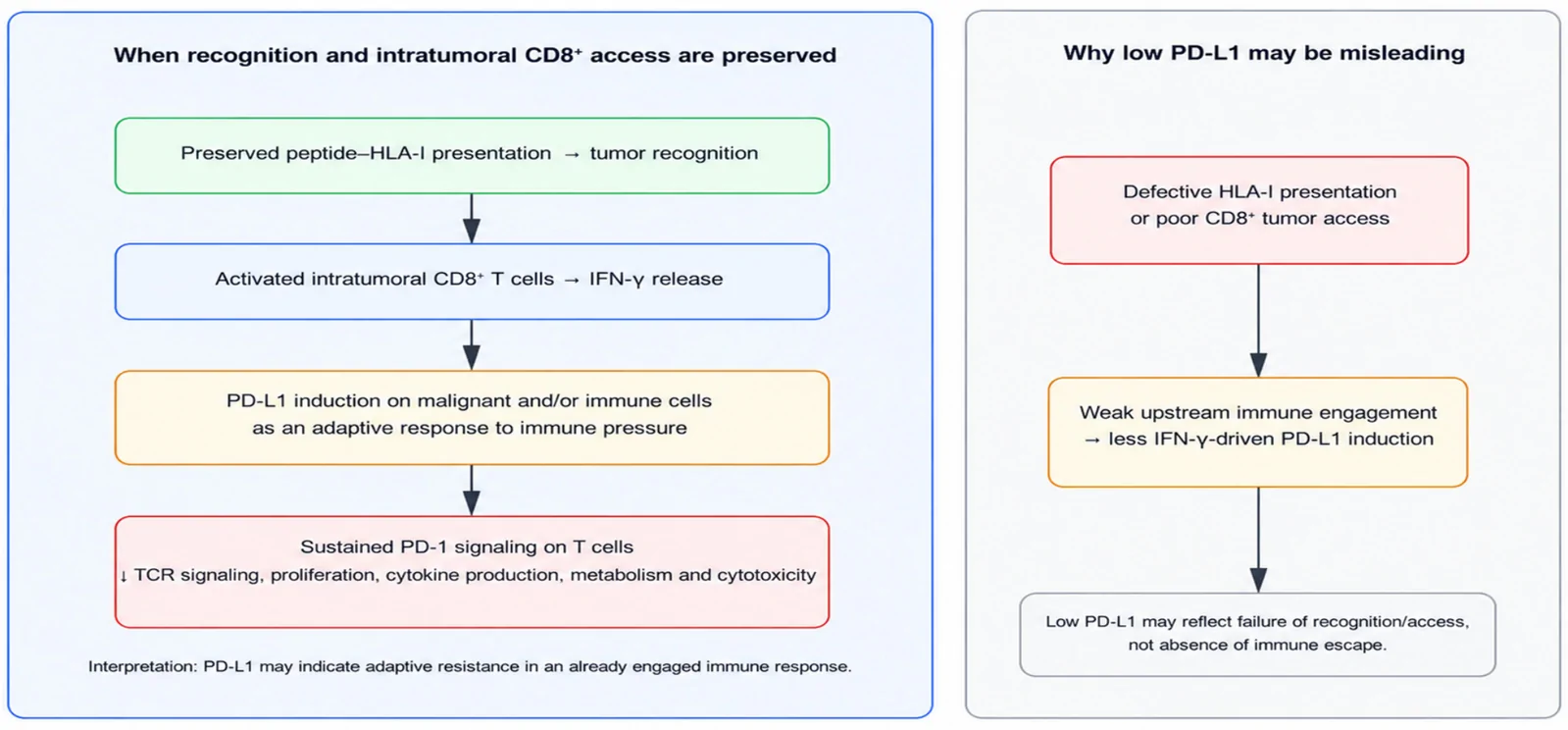
**Context-dependent interpretation of PD-1/PD-L1 signaling in cervical cancer.** When HLA-I-mediated tumor recognition and intratumoral CD8^+^ T-cell access are preserved, IFN-γ released by activated T cells can induce PD-L1 expression on malignant and immune cells, resulting in adaptive checkpoint-mediated suppression. Conversely, low PD-L1 expression in HLA-I-deficient or poorly infiltrated tumors may reflect weak upstream immune engagement rather than absence of immune escape.

This dual role helps explain why PD-L1 has performed inconsistently as a stand-alone biomarker. The same PD-L1 result may represent biologically different states. In a tumor with preserved HLA-I expression and intratumoral CD8^+^ infiltration, PD-L1 expression is compatible with adaptive resistance arising in response to ongoing immune recognition. As illustrated in Figure 4, the biological significance of PD-1/PD-L1 signaling depends on whether antigen presentation and CD8^+^ T-cell access are preserved, distinguishing adaptive checkpoint-mediated resistance from tumors in which immune escape occurs primarily through upstream failure of recognition or immune exclusion. By contrast, low PD-L1 expression in a poorly infiltrated or HLA-I-deficient tumor may reflect failure to generate the upstream immune response required for adaptive PD-L1 induction rather than the absence of immune escape [11,30]. Cellular localization adds another layer of uncertainty, because similar overall staining may represent expression predominantly by malignant cells in one tumor and by immune cells in another.

Sustained PD-1 signaling reduces T-cell receptor signaling, proliferation, cytokine production, metabolic activity, and cytotoxic function [25,30]. In cervical cancer, however, the more relevant question is whether PD-1-mediated inhibition is the dominant barrier to tumor control. Releasing this checkpoint is biologically most plausible when tumor recognition is preserved and CD8^+^ T cells have already reached the malignant compartment. It is less likely to compensate for an upstream loss of peptide–HLA-I presentation or for a tumor in which cytotoxic lymphocytes remain largely excluded from tumor nests. Checkpoint dependence therefore cannot be inferred from PD-L1 staining alone.

Clinical findings are consistent with this interpretation. Cemiplimab has produced benefit in both PD-L1-positive and PD-L1-negative cervical cancers, indicating that assay-defined PD-L1 status does not fully capture dependence on the pathway [31]. PD-L1 expression is also heterogeneous within individual tumors, differs between tumor and immune-cell compartments, and varies across histological subtypes [32]. Meta-analytic data have associated higher PD-L1 expression with poorer overall survival, but the association has been less consistent for progression-free survival, and comparisons are complicated by differences in antibody selection, scoring systems, cellular compartments, and cut-offs [33].

PD-L1 should therefore not be treated as a self-contained immune phenotype. Its interpretation becomes more informative when placed downstream of antigen presentation and considered together with the spatial distribution and functional state of CD8^+^ T cells. In an HLA-I-preserved, T-cell-infiltrated tumor, PD-L1 may indicate an established antitumor response that has encountered a potentially reversible inhibitory barrier. In an HLA-I-deficient or immune-excluded tumor, checkpoint signaling may be secondary to a more fundamental failure of recognition or access. From this perspective, the value of PD-L1 lies less in its isolated expression level than in identifying where checkpoint-mediated inhibition operates within the broader sequence of cervical cancer immune escape.

## 8. CTLA-4 Pathway

CTLA-4 mainly regulates T-cell priming and early clonal expansion by competing with CD28 for binding to CD80/CD86 on antigen-presenting cells [11,34]. In cervical cancer, this is relevant because failure of antitumor immunity may occur before effector T cells reach the tumor. Even when HPV-derived antigens are available, an insufficiently primed or poorly expanded T-cell response may limit the development of effective tumor-directed immunity.

The role of CTLA-4 is also closely linked to FOXP3^+^ regulatory T cells. Tregs constitutively express CTLA-4 and use this pathway as part of their suppressive activity [26,27,34]. CTLA-4 therefore connects two levels of immune regulation: it can restrict the expansion of newly activated effector T cells while also reinforcing suppression by the regulatory compartment. In a cervical tumor already enriched in FOXP3^+^ Tregs, these effects may substantially reduce the strength of an otherwise antigen-specific response.

The importance of this pathway, however, depends on whether tumor recognition is still possible. When HLA-I-mediated presentation of HPV-derived peptides is preserved, impaired priming or regulatory suppression may become an important barrier to effective immunity. In contrast, if antigen presentation is profoundly disrupted, increasing T-cell activation alone is unlikely to restore tumor control because the malignant cells may no longer be adequately recognized. CTLA-4-mediated suppression should therefore be interpreted in the context of the upstream integrity of antigen presentation.

This distinction is also relevant when considering CTLA-4 as a therapeutic target. Its contribution is likely to be greatest in tumors in which antigen recognition remains intact but the generation or expansion of an effective T-cell response is constrained. It is less likely to represent the dominant resistance mechanism when immune escape is primarily driven by HLA-I loss or by marked exclusion of effector cells from tumor nests.

**Figure 5:**
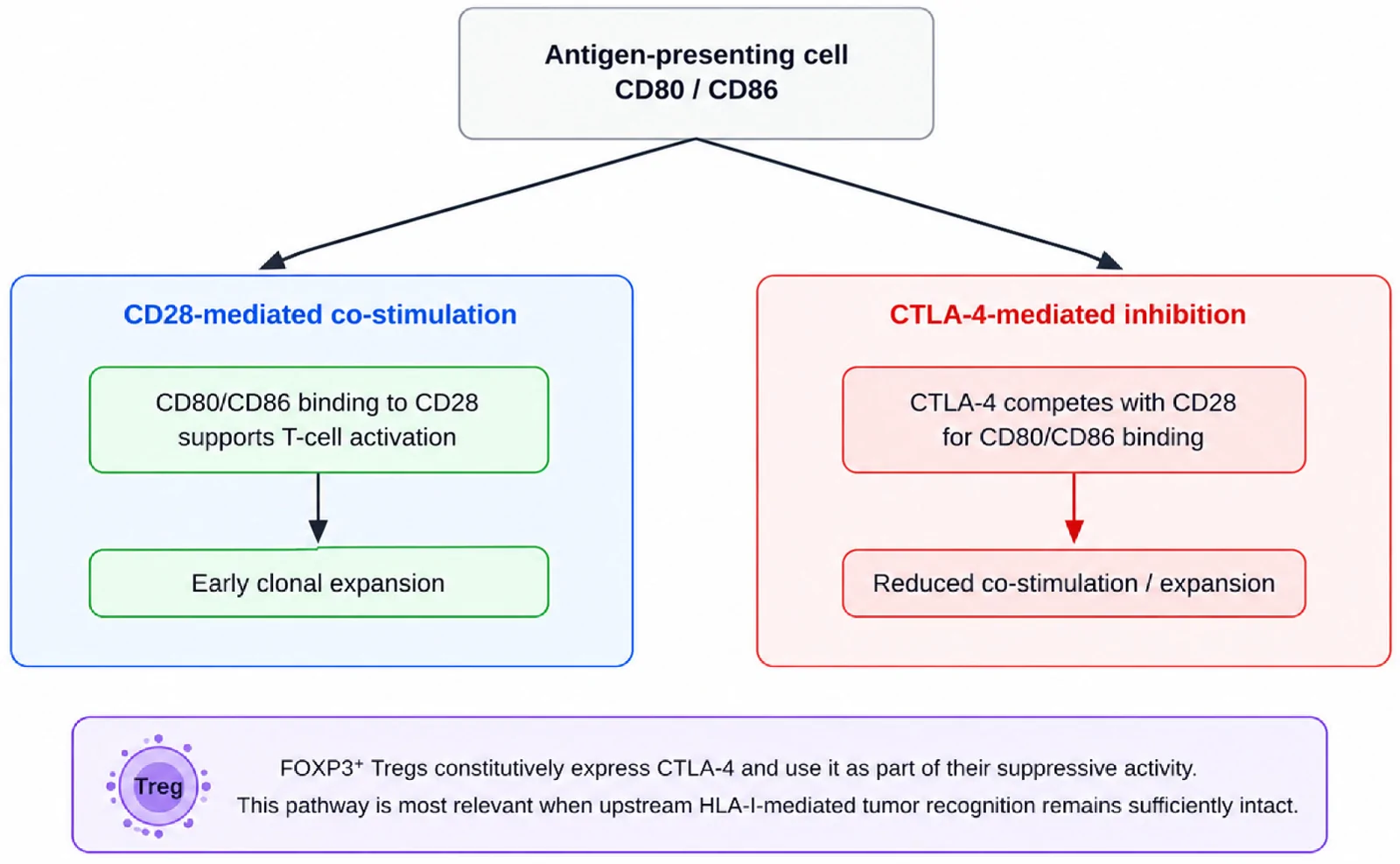
**CTLA-4-mediated regulation of T-cell priming and early clonal expansion.** CD28 engagement by CD80/CD86 provides co-stimulatory signaling that supports T-cell activation and expansion, whereas CTLA-4 competes with CD28 for CD80/CD86 binding and reduces co-stimulation. FOXP3^+^ regulatory T cells constitutively express CTLA-4 and use this pathway as part of their suppressive activity.

Within the integrated model proposed here, CTLA-4 is therefore best viewed as a regulator of the magnitude and quality of an antigen-specific T-cell response rather than as a mechanism that determines whether the tumor is recognized in the first place. As illustrated in Figure 5, CTLA-4-mediated inhibition operates primarily at the level of T-cell priming and regulatory suppression, and its biological impact depends on the preservation of upstream antigen recognition. Its biological relevance depends on the immune context in which it operates, particularly the integrity of HLA-I-mediated antigen presentation and the extent of FOXP3^+^ Treg-mediated suppression.

## 9. Integrated Immune Escape Model

Persistent E6 and E7 expression supplies cervical tumors with recognizable viral antigens. In an intact response, those antigens are processed, displayed by HLA-I, recognized by CD8^+^ T cells, and followed by elimination of the transformed cell. Cervical carcinogenesis disrupts this sequence at several points rather than through one dominant pathway [11,16,17].

Loss or downregulation of HLA-I acts upstream by reducing the peptide–HLA complexes available for recognition. Viral antigens can therefore remain abundant while the tumor becomes poorly visible to CD8^+^ T cells [16,17,18].

In tumors that remain recognizable, chronic stimulation and IFN-γ-driven PD-L1 expression can turn an initially active immune response into adaptive resistance. Repeated PD-1 signaling then reduces cytokine production and cytotoxicity and promotes exhaustion [11,25,30].

FOXP3^+^ regulatory T cells add a further layer of suppression through inhibitory cytokines, CTLA-4-dependent effects on antigen-presenting cells, and restriction of effector-cell expansion [26,27,34]. Their impact depends on density, functional phenotype, and proximity to tumor-reactive lymphocytes.

**Figure 6:**
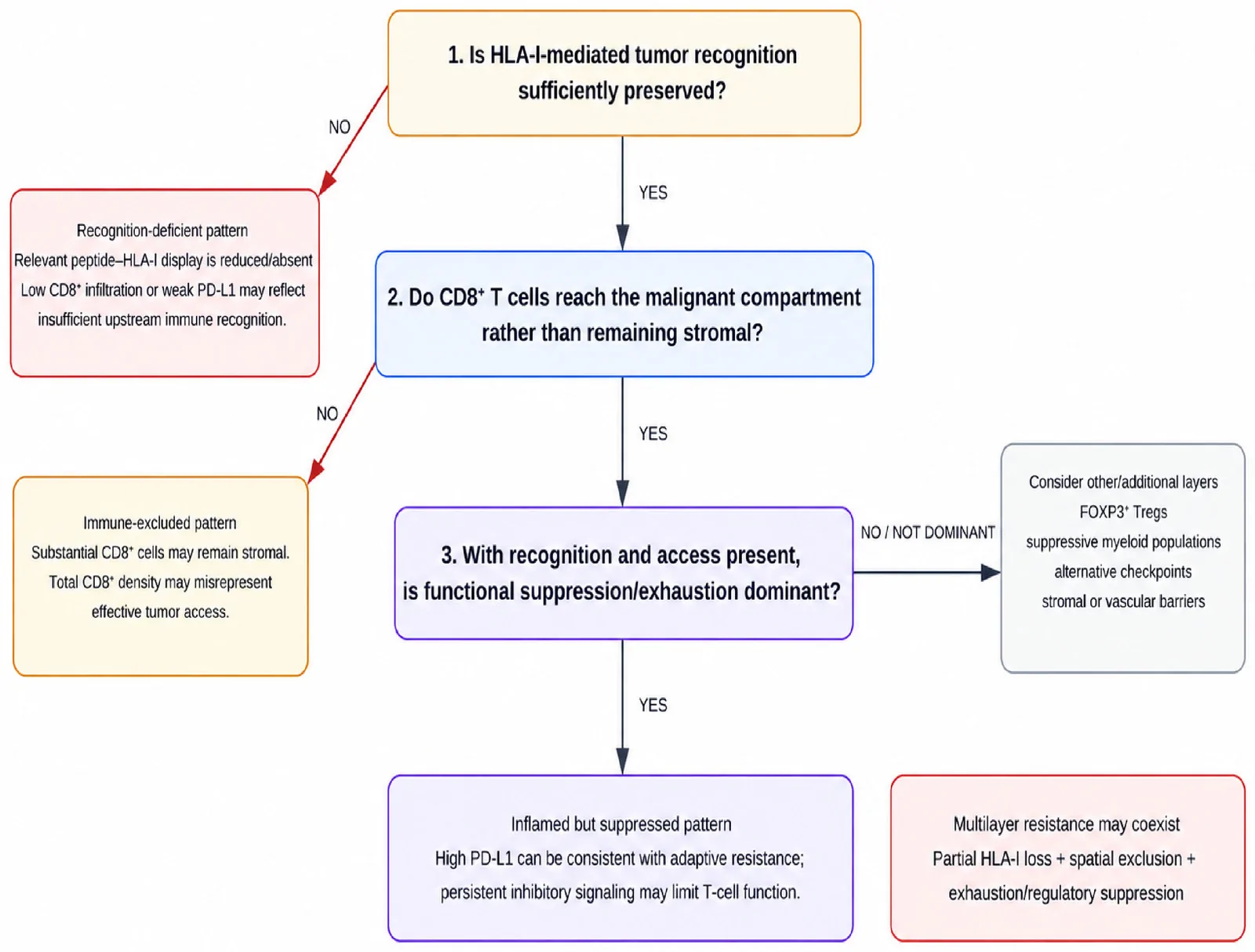
**Mechanistic decision framework for interpreting immune escape in cervical cancer.** The proposed model evaluates immune resistance sequentially according to HLA-I-mediated tumor recognition, spatial access of CD8^+^ T cells to the malignant compartment, and functional suppression or exhaustion when recognition and access are preserved. Defective antigen presentation is consistent with a recognition-deficient state, preserved recognition with stromal restriction of CD8^+^ cells defines an immune-excluded state, and preserved recognition and access with inhibitory T-cell dysfunction is compatible with an inflamed but suppressed state. These mechanisms may coexist as multilayer resistance. The framework is conceptual and is not intended as a validated clinical classifier.

The resulting immune-escape phenotype is not merely the sum of four markers. HLA-I loss can render checkpoint release irrelevant [16,17,18,19,20]; stromal exclusion can separate otherwise competent CD8^+^ cells from their targets [24,28]; and regulatory suppression can persist after PD-1 blockade [26,27,30,34]. Figure 6 integrates these dependencies and shows why no single biomarker adequately describes the cervical tumor immune microenvironment [11,13].

## 10. Therapeutic Implications

These mechanisms suggest several therapeutic entry points, but they also impose a useful constraint: a treatment can work only if the remaining steps of the cancer–immunity cycle are sufficiently intact. This helps explain both the success of checkpoint inhibition and the persistence of primary and acquired resistance.

### 10.1. Restoring T-Cell Function: Targeting the PD-1/PD-L1 Pathway

Blocking PD-1/PD-L1 can recover part of the function of exhausted T cells and has changed systemic therapy for persistent, recurrent, and metastatic cervical cancer [11,25,30]. Its clinical success is the strongest evidence that immune suppression is therapeutically reversible in at least a subset of patients.

Pembrolizumab is the best-established example. When added to platinum-based chemotherapy, with or without bevacizumab, it improved progression-free and overall survival in PD-L1-positive persistent, recurrent, or metastatic cervical cancer and entered first-line treatment [35,36].

Moreover, pembrolizumab is now used to treat high-risk, locally advanced diseases with the goal of curing them. Pembrolizumab was added to concurrent chemoradiotherapy and continued as maintenance in KEYNOTE-A18, which increased progression-free survival and supported regulatory approval in 2024 [37]. A significant overall-survival improvement was later shown by the mature analysis: 36-month survival was 82.6% with pembrolizumab compared to 74.8% with placebo (hazard ratio for death, 0.67; *p* = 0.0040) [38]. Although the outcome reflects pembrolizumab in conjunction with final chemoradiotherapy rather than checkpoint blocking alone, this was a significant extension of checkpoint therapy into non-metastatic cervical cancer.

Cemiplimab also improved overall survival compared with investigator’s-choice chemotherapy after platinum treatment. Benefit was observed in both PD-L1-positive and PD-L1-negative tumors, an important reminder that assay-defined PD-L1 status does not fully capture checkpoint dependence [31,39].

Nevertheless, most patients do not obtain durable control from PD-1 blockade alone. Absent peptide–HLA presentation, stromal exclusion, terminal T-cell dysfunction, and FOXP3^+^ or myeloid suppression can all preserve resistance after the receptor is blocked [11,16,25,30]. Combination therapy should therefore be matched to an identified complementary defect rather than justified solely by the number of pathways targeted.

### 10.2. Enhancing Early T-Cell Activation: CTLA-4 Blockade

CTLA-4 blockade addresses priming and clonal expansion rather than the same biological step as PD-1 inhibition. This complementary mechanism provides a rationale for dual blockade, but mechanistic complementarity does not itself establish a favorable clinical risk–benefit balance [34].

Ipilimumab monotherapy has shown limited activity in cervical cancer. Combining CTLA-4 and PD-1 inhibition may broaden T-cell priming while restoring exhausted effectors, but it also increases immune-related toxicity and complicates attribution of benefit [34].

CheckMate 358 reported durable activity with nivolumab plus ipilimumab in recurrent or metastatic cervical cancer [40]. The study supports further evaluation, but its open-label, non-randomized cohort does not determine whether dual blockade is superior to PD-1-directed therapy or which patients justify the added toxicity.

CTLA-4 inhibition should therefore be viewed as an investigational component of combination therapy, not an established alternative to PD-1 blockade. Randomized comparisons and biomarker-linked analyses are needed before its place in routine cervical cancer treatment can be defined.

### 10.3. Restoring Antigen Recognition

Checkpoint inhibition cannot reactivate a response that never reaches tumor recognition. Its efficacy still depends on processing and presentation of HPV-derived antigens through HLA-I, making antigen-presentation defects a plausible cause of primary or acquired resistance [16,17,18].

Therapeutic HPV vaccines approach this problem by expanding E6- and E7-specific T-cell populations. That can increase the supply of tumor-reactive effectors, but it cannot by itself correct structural HLA-I loss or guarantee trafficking into tumor nests [41].

Combining vaccination with checkpoint inhibition is therefore biologically coherent when inadequate priming and reversible T-cell inhibition coexist. The same combination is less likely to succeed when the dominant lesion is irreversible antigen-presentation loss or strong stromal exclusion [11,41].

The translational task is to distinguish those situations before treatment. Without mechanism-based selection, a negative combination trial may reflect enrollment of biologically incompatible tumors rather than failure of the therapeutic concept itself.

### 10.4. Targeting the Immunosuppressive Tumor Microenvironment

Local immune suppression remains a barrier even when HPV-specific T cells are present and capable of recognition. Regulatory lymphocytes, suppressive myeloid populations, abnormal vasculature, inhibitory cytokines, and physical stromal barriers can each prevent an activated T cell from reaching or killing a malignant cell [11,26,27].

For instance, a parallel pathway is provided by suppressive myeloid cells. SERPINB3-associated chemokines increased MDSC and TAM recruitment, reduced cytotoxic T-cell activity, and increased radiation resistance in cervical carcinoma models and patient material [42]. Additionally, cervical tumor-infiltrating lymphocytes can co-express PD-1 with TIGIT, TIM-3, LAG-3, and NKG2A, indicating that T-cell dysfunction is not limited to PD-1 and CTLA-4 [43]. Although these results point to potential mechanisms of residual suppression following PD-1 inhibition, neither their prognostic usefulness nor the therapeutic advantage of targeting them in cervical cancer have been proven.

Current evidence suggests that modulation of the tumor microenvironment is most effective when incorporated into combination treatment strategies. The addition of chemotherapy or radiotherapy to immune checkpoint inhibitors may enhance antitumor immunity by increasing tumor antigen release, promoting immune-cell infiltration, and reducing local immunosuppression. Similarly, anti-angiogenic agents may improve immune-cell trafficking by normalizing the tumor vasculature, thereby creating a microenvironment that is more permissive to effective immune responses [11]. Therapeutic HPV vaccines may further complement these approaches by expanding HPV-specific cytotoxic T-cell populations, providing additional immune effectors capable of recognizing transformed cervical epithelial cells.

The rationale for combination treatment is therefore strong but not self-validating. Much of the evidence for proposed regimens remains early-phase or non-randomized, and any gain in activity must be weighed against toxicity, cost, sequencing uncertainty, and the risk of treating resistance mechanisms that are not present in a given tumor [11].

### 10.5. Integrated Immune Profiling for Patient Stratification

No single immune marker captures the heterogeneity of cervical cancer. PD-L1 is clinically actionable in defined settings, but response varies within each expression category, and neither lymphocyte density nor HLA-I status is sufficient when interpreted without spatial and functional context [11,30].

Integrated profiling seeks to combine antigen-presentation status, immune-cell composition, checkpoint expression, and spatial organization. Its value would lie in identifying the dominant resistance pattern in an individual tumor, not simply in producing a larger panel of correlated measurements [11,13,24]. Table 1 summarizes the principal candidate components.

Spatial organization may add information that cell density misses. A tumor with intratumoral CD8^+^ cells is biologically different from one in which a similar number of cells remains confined to the stroma, and FOXP3^+^ cells may have different implications depending on their proximity to effector cells and malignant nests [28]. These observations are hypothesis-generating, however, and do not yet define a reproducible clinical classifier.

Integrated scores introduce their own statistical risks. The number of candidate features can be large relative to the number of outcome events, encouraging overfitting, unstable feature selection, and optimistic performance estimates. Correlated markers, data-driven cut-offs, missing tissue regions, and reuse of the same cohort for model development and testing further weaken apparent accuracy. A clinically credible model will require prespecified variables and endpoints, transparent handling of missing data, internal validation that accounts for optimism, independent external validation, calibration, and proof that it adds decision-relevant information beyond stage, histology, and established biomarkers. Until then, integrated immune profiles remain research tools rather than validated treatment-selection assays.

### 10.6. Mechanistic Stratification of Immune Escape and Translational Barriers

One reason immune biomarkers have produced conflicting results in cervical cancer is that they describe different stages of the antitumor response. HLA-I expression reflects whether tumor cells can present HPV-derived peptides and remain visible to CD8^+^ T cells. CD8^+^ infiltration indicates whether effector cells are present, but not necessarily whether they have entered tumor nests or remain functional. PD-L1, in turn, may reflect an active immune response that has triggered adaptive inhibition rather than a uniformly suppressive tumor phenotype. These markers therefore become difficult to interpret when considered separately [20,29,31,32,33].

A more useful way to approach this heterogeneity is to ask where the immune response is failing. The first question is whether tumor recognition is still possible. When HLA-I expression is substantially reduced or the antigen-processing machinery is disrupted, HPV-derived antigens may remain present without being effectively displayed to cytotoxic T cells. In this setting, low CD8^+^ infiltration or weak PD-L1 expression should not automatically be interpreted as evidence of a relatively inactive checkpoint pathway. They may instead reflect the absence of sufficient upstream immune recognition. This distinction is particularly important for structural HLA-I defects, which are unlikely to be corrected by simply releasing T-cell inhibition [19,20].

If antigen presentation is preserved, the next question is whether CD8^+^ T cells can actually reach malignant cells. Some cervical tumors contain substantial numbers of cytotoxic lymphocytes, but these cells remain concentrated in the surrounding stroma rather than within tumor nests. Such tumors are biologically different from truly inflamed tumors despite potentially similar total CD8^+^ counts. In this situation, the major barrier may be spatial exclusion rather than a lack of immune recruitment. Checkpoint inhibition may have limited effect if the relevant effector cells cannot gain access to their targets [20,29].

A third pattern emerges when tumor recognition is preserved and CD8^+^ T cells are already present within the malignant compartment. Here, persistent antigen exposure and local inhibitory signaling become more plausible explanations for treatment failure. High PD-L1 expression in this setting can be interpreted as evidence of adaptive resistance: the tumor has been recognized, an immune response has developed, and inhibitory signaling has subsequently limited its effectiveness. This is biologically different from a poorly infiltrated or HLA-I-deficient tumor in which low PD-L1 may simply reflect failure to generate the inflammatory signals that normally induce its expression. The observation that cemiplimab can benefit both PD-L1-positive and PD-L1-negative cervical cancers further illustrates why PD-L1 status alone cannot identify the dominant resistance mechanism [31,32,33].

These patterns are unlikely to remain completely separate in individual tumors. Partial HLA-I loss may coexist with stromal exclusion, T-cell exhaustion, regulatory-cell activity, suppressive myeloid populations, or alternative checkpoints. Such multilayer resistance is probably one reason why combinations that appear mechanistically rational do not necessarily produce additive clinical benefit. Therapeutic vaccination may increase the number of HPV-specific T cells without improving their access to tumor nests; checkpoint blockade may restore part of the function of exhausted cells without correcting loss of antigen presentation; and reducing regulatory suppression may have little effect when other stromal or myeloid barriers remain intact [11,19,20,41].

Viewed in this way, PD-L1 should not be interpreted as an isolated positive or negative biomarker. Its meaning depends on what has happened earlier in the immune response. PD-L1 expression in a tumor with preserved HLA-I and intratumoral CD8^+^ infiltration is consistent with an established immune response that has encountered an inhibitory barrier. By contrast, low PD-L1 expression in a tumor with defective HLA-I presentation or marked immune exclusion may indicate failure of immune engagement rather than absence of immune escape. This provides a biological explanation for some of the apparently contradictory associations reported for PD-L1 in cervical cancer.

We therefore propose a simple mechanistic hierarchy for interpreting the cervical tumor immune microenvironment. The first step is to establish whether HLA-I-mediated tumor recognition is sufficiently preserved. If it is, the second is to determine whether CD8^+^ T cells reach the malignant compartment or remain spatially excluded. Only when recognition and access are both present does it become meaningful to ask whether exhaustion and checkpoint-mediated suppression represent the major remaining barrier. FOXP3^+^ regulatory T cells and other suppressive populations can then be considered as additional layers that modify this sequence rather than as independent explanations of resistance.

This framework is not intended as a validated clinical algorithm, and individual tumors may move between these states during treatment. Its purpose is to shift interpretation away from isolated biomarker values toward the biological sequence required for an effective antitumor response. Prospective studies should therefore test whether combining HLA-I status, spatial CD8^+^ localization, and functional markers of T-cell suppression can identify the dominant resistance mechanism more reliably than any of these variables alone. Paired tumor sampling will be particularly important, since treatment-related immune remodeling has already been demonstrated in cervical cancer and cannot be reconstructed reliably from a single pretreatment biopsy [44].

## 11. Future Directions

The field now needs studies designed around unresolved clinical questions rather than further catalogues of immune markers. Three priorities stand out: measuring how immune states change during treatment, testing therapeutic vaccines in cohorts that represent HLA and HPV diversity, and validating analytical tools in prospective multicenter settings. These priorities should also be addressed in populations that reflect the global burden of cervical cancer, particularly women living with HIV and patients from high-burden low- and middle-income countries, who remain incompletely represented in the available evidence [45,46,47].

### 11.1. Multiplex and Spatial Immune Profiling

Multiplex immunohistochemistry, imaging mass cytometry, and spatial transcriptomics can characterize immune-cell composition while preserving tissue architecture [48,49]. This distinction is important in cervical cancer because tumors with similar numbers of CD8^+^ cells may be biologically different when lymphocytes enter malignant nests in one case but remain confined to the surrounding stroma in another. Most available datasets, however, remain cross-sectional and cannot establish whether one spatial phenotype evolves into another.

Computational trajectory analysis provides only a partial solution. Pseudotime orders cells according to transcriptional similarity, but it does not demonstrate that the same cells or clones have actually progressed through those states. This is particularly problematic in the heterogeneous cervical tumor microenvironment, where similar transcriptional profiles may arise in different anatomical niches or from unrelated T-cell clonotypes. Conversely, cells belonging to the same clone may adopt different activation or exhaustion states depending on their local environment [49,50]. Pseudotime should therefore be treated as a hypothesis about cellular relationships rather than as a substitute for longitudinal observation.

A stronger approach would combine repeated tissue sampling with clonotype tracking. Matched biopsies obtained before treatment, during therapy, and at response or progression should include multiple tumor regions whenever feasible. Single-cell RNA sequencing can define functional T-cell states, T-cell-receptor sequencing can determine whether the same clonotypes persist or expand over time, and spatial profiling can establish whether those clones remain stromal or gain access to malignant nests. Recent cervical cancer studies illustrate the value of combining these modalities and have linked oligoclonal CD8^+^ T-cell revival with response to neoadjuvant chemoimmunotherapy [50,51].

Evidence of genuine clonal revival should therefore require more than an increase in post-treatment CD8^+^ cell density. A more convincing signal would include expansion of the same TCR clonotype across time, a concordant shift toward an activated cytotoxic transcriptional state, and evidence that the clone is present within the tumor compartment. If such a change is detected only in one sampled region, spatial heterogeneity remains an alternative explanation. Repeated multiregion sampling is therefore necessary to distinguish treatment-associated immune remodeling from differences between successive biopsy sites.

Analytically, these data should be evaluated at the level of the patient, region, and time point rather than treating individual cells as independent observations. Concordance between clonotype dynamics, transcriptional state, spatial localization, and HLA-I/PD-L1 context would provide stronger evidence of a true biological transition than any modality alone. The aim is not to construct the most complex trajectory possible, but to determine whether the same immune response can be followed through time and space and thereby distinguish genuine T-cell revival from spatial sampling variance [44,50,51].

### 11.2. Therapeutic HPV Vaccines

Therapeutic HPV vaccines are attractive because E6 and E7 are retained in transformed cells and are absent from normal tissue. DNA, RNA, peptide, protein, viral-vector, bacterial-vector, and dendritic-cell platforms have all been used to induce HPV-specific cellular immunity [41,52]. The central question is no longer whether a vaccine can generate an immune response, but whether that response reaches and controls invasive tumors in clinically representative patients.

The existing clinical cohorts do not answer that question uniformly. The randomized phase 2b VGX-3100 trial demonstrated histologic regression in HPV16/18-positive CIN2/3, but a precancerous lesion with an intact treatment window is not equivalent to recurrent or metastatic cervical cancer [53]. Conversely, the phase 2 study of ISA101 with nivolumab enrolled a small, single-arm group with different incurable HPV16-positive tumor sites, making it difficult to separate vaccine activity from checkpoint blockade or to generalize the result specifically to cervical cancer [54]. Across platforms, differences in disease stage, prior therapy, HPV type, immune endpoints, and clinical endpoints make apparently encouraging results difficult to compare.

HLA diversity is an additional, underexamined source of heterogeneity. A T-cell response requires vaccine-derived peptides to bind the patient’s HLA molecules, and cervical cancer data indicate that HPV16 E6-specific responses and clinical outcome vary with HLA-A alleles [55]. Long-peptide, DNA, RNA, and vector platforms can provide multiple epitopes and are less narrowly restricted than a single short peptide, but they do not eliminate HLA dependence because antigen processing and presentation still occur in the host. Most therapeutic-vaccine trials were not designed or powered for HLA-stratified efficacy analyses, and geographically concentrated recruitment may underrepresent allele frequencies present in other populations. Immunogenicity observed in one cohort should therefore not be assumed to be universal.

Future trials should incorporate prespecified HLA typing, report cellular responses by HLA allele or supertype, recruit across geographically and ancestrally diverse populations, and distinguish failure of immune priming from failure caused by tumor HLA-I loss. Multi-epitope and multi-genotype constructs may improve population coverage, but they still require prospective evidence of clinical benefit. Randomized studies with comparable disease settings and meaningful clinical endpoints—not immunogenicity alone—will be necessary before therapeutic HPV vaccines can be placed within routine multimodal treatment [41,52,53,54,55].

### 11.3. Emerging Cellular Immunotherapies

Cellular therapy offers a different way to increase the number or specificity of tumor-reactive effectors. Rather than relying on endogenous priming alone, tumor-infiltrating lymphocytes (TILs) or genetically engineered T cells can be selected and expanded outside the patient [11,56].

HPV-targeted TIL therapy has produced durable complete regressions in selected patients with metastatic cervical cancer [56]. These responses establish proof of principle, but they arose in highly selected patients treated at specialized centers and should not be read as an estimate of population-level efficacy.

T-cell-receptor-engineered and chimeric antigen receptor T cells are also being investigated. For TCR-engineered products, HLA restriction and antigen-presentation loss are particularly relevant; CAR-T cells avoid HLA restriction but require a suitable surface target and must still overcome trafficking and local immune suppression [11,57].

Manufacturing time, cost, lymphodepletion, toxicity, and access currently limit these therapies to expert centers. Their broader role will depend as much on scalable delivery and rational patient selection as on further improvements in cell engineering.

### 11.4. Artificial Intelligence and Digital Pathology

The most relevant role of artificial intelligence in this setting is not simply to reproduce conventional histological classification. Its greater potential lies in extracting spatial information from tissue and relating that information to biologically defined mechanisms of immune escape. In cervical cancer, this could include quantitative assessment of HLA-I expression, separation of tumor- and immune-cell PD-L1 staining, localization of CD8^+^ and FOXP3^+^ cells, and measurement of their relationship to malignant nests. The difficulty is that these measurements are only as reliable as the tissue compartments on which they are based [58,59].

Spatial segmentation is therefore an important computational bottleneck. Distinguishing malignant epithelium from surrounding stroma, inflammatory aggregates, necrosis, and non-neoplastic tissue may appear straightforward to a pathologist, but these boundaries are not necessarily reproduced consistently by an algorithm. This becomes especially important when the biological question depends on whether CD8^+^ cells are located within tumor nests or remain outside them. A modest error in defining the tumor–stroma interface can change a tumor from apparently infiltrated to apparently excluded. The problem is amplified across institutions because staining protocols, image preprocessing, scanner characteristics, resolution, and tissue quality differ between laboratories, creating domain shifts that can impair model generalization [58,59].

The challenge is not solved simply by increasing the size of the image dataset. A model can learn reproducible visual patterns while still learning the wrong biological target. This is particularly relevant for immune profiling because many clinically important immune states are not directly visible on routine morphology. Current cervical pathology AI systems remain largely dependent on visual image features and have limited ability to capture molecular changes or dynamic features of the tumor microenvironment [59]. A lymphocyte-rich region, for example, does not reveal whether the infiltrating CD8^+^ cells recognize HPV-derived peptide–HLA complexes, and morphology alone cannot establish whether preserved lymphocyte infiltration represents effective immunity or functionally exhausted T cells.

For this reason, moving from morphological classification to functional immune-escape phenotyping requires a different definition of ground truth. Rather than training an algorithm only to reproduce a pathologist-assigned morphological category, the target phenotype should be anchored to independent biological measurements. In the framework proposed in this review, image-derived spatial features could be integrated with HLA-I, PD-L1, and immune-cell phenotyping to distinguish tumors in which recognition is impaired from those in which effector cells are spatially excluded or present but functionally suppressed. Multiplex imaging and spatial or single-cell molecular profiling may provide the orthogonal information needed to define such phenotypes [48,50].

This distinction also exposes an important limitation of apparently high model performance. A classifier may achieve excellent discrimination within a single cohort because staining, scanners, specimen preparation, and patient selection are relatively uniform. Such performance does not establish that the model has learned a transferable immune phenotype. Digital pathology studies frequently provide incomplete information about case selection, data partitioning, and reference standards, while external validation remains inconsistent [58]. In cervical pathology specifically, differences in laboratory protocols, staining procedures, imaging systems, and preprocessing continue to limit generalization between institutions [59]. Evaluation should therefore test not only the final patient-level prediction but also whether the intermediate measurements—tumor segmentation, immune-cell localization, and compartment-specific biomarker quantification—remain stable when the model is transferred to another cohort.

Multimodal modeling introduces a further difficulty. Combining morphology, immunohistochemistry, molecular assays, and spatial data can improve biological resolution, but it also increases data heterogeneity and creates a model that may depend on assays that are unavailable for many patients. The objective should therefore not be maximal data integration for its own sake. A useful strategy would first use high-dimensional datasets to define biologically credible immune-escape phenotypes and then determine whether a smaller set of reproducible image-derived and routinely measured features can recover those phenotypes in clinical material. In this sense, the relevant computational problem is one of phenotype translation rather than simple image classification.

For cervical cancer, the most informative AI application would therefore be one that follows the biological sequence proposed in this review: whether malignant cells remain recognizable through HLA-I, whether CD8^+^ T cells gain access to tumor nests, and whether an infiltrated response is subsequently constrained by regulatory or checkpoint-mediated suppression. Such a model would move digital pathology beyond morphological pattern recognition toward mechanistically interpretable immune phenotyping. Its success should ultimately be judged not by whether it predicts an outcome from an image, but by whether it can reproducibly identify the dominant immune-escape state across patients, tissue regions, and clinical settings [58,59].

## 12. Limitations

The main limitation of this review lies in the heterogeneity of the evidence on which the proposed integrated model is based. Studies of cervical cancer immunity differ considerably in patient population, disease stage, histological subtype, sample size, previous treatment, and analytical design. Findings that appear biologically contradictory may therefore sometimes reflect differences between cohorts rather than genuinely opposing immune mechanisms [11,13,24].

This problem is compounded by the lack of uniformity in biomarker assessment. Studies of HLA-I have used different antibodies, scoring approaches, and definitions of preserved or reduced expression [16,17,18,19,20]. Similar variability affects PD-L1, for which antibody clones, cellular compartments, scoring systems, and positivity thresholds are not interchangeable [32,33]. The literature on CD8^+^ and FOXP3^+^ cells is likewise influenced by differences in tissue selection, tumor-versus-stroma definitions, counting methods, and cut-offs [24,26,27,28,29]. These methodological differences make direct comparison between studies difficult and limit the extent to which apparently consistent associations can be treated as reproducible biomarkers.

A further concern is that much of the available biomarker evidence is retrospective. Many studies were designed to identify associations with outcome rather than to determine whether a particular immune feature predicts benefit from a specific treatment. Prognostic and predictive effects therefore remain difficult to separate, particularly when treatment exposure and disease stage differ across cohorts [11,24,30]. This distinction is important for the integrated framework proposed here, because a biologically plausible marker is not necessarily a clinically useful treatment-selection marker.

Spatial and temporal sampling introduce another source of uncertainty. A single biopsy captures only one region of a heterogeneous tumor at one point in its evolution. It may therefore misrepresent HLA-I expression, immune-cell localization, checkpoint activity, or the balance between effector and regulatory populations elsewhere in the lesion. Treatment adds a further dimension, because radiochemotherapy and immunotherapy can remodel both the composition and functional state of the tumor microenvironment. Paired analyses in cervical cancer have already shown that such changes may not be predictable from pretreatment tissue alone [44]. Integrated immune profiles based on static samples should therefore be interpreted cautiously until their spatial and temporal stability has been established [11,13,24,30,44].

Generalizability is also an important limitation of the current evidence base. Women living with HIV carry a substantially greater burden of cervical cancer, while HIV-associated changes in cervical mucosal immunity may alter the tumor–immune relationships described in predominantly HIV-unselected cohorts [45,46]. At the same time, patients from many low- and middle-income countries, where the burden of cervical cancer is greatest, remain inadequately represented in immunotherapy research. Differences in trial participation, access to treatment, pathology infrastructure, and availability of complex biomarker platforms may further limit the transfer of findings from highly resourced research settings into routine clinical practice [47].

These limitations do not invalidate an integrated view of cervical cancer immune escape, but they define how it should be used. At present, the proposed framework should be regarded as a biologically informed model for organizing existing evidence rather than as a validated clinical classifier. Its value will ultimately depend on prospective studies using standardized assays; spatially and temporally informative sampling; representative patient populations; and predefined analyses that test whether integration of HLA-I status, immune-cell localization, and functional immune suppression provides information beyond established clinicopathological variables.

## 13. Conclusions

Cervical cancer presents a clear immunological paradox: E6 and E7 provide persistent, tumor-associated viral antigens, yet malignant cells can remain beyond effective immune control. The explanation lies in a sequence of interacting failures—reduced HLA-I presentation, poor access or exhaustion of CD8^+^ T cells, regulatory suppression, and PD-1/PD-L1 and CTLA-4 signaling—rather than in one dominant pathway.

This integrated view also explains why individual biomarkers and single-agent interventions have uneven performance. PD-L1 can mark adaptive resistance or an active immune response; abundant CD8^+^ cells can be excluded or dysfunctional; and checkpoint blockade cannot restore recognition after irreversible HLA-I loss. A rational combination should therefore address a demonstrated complementary defect, not merely combine agents with plausible mechanisms.

The immediate clinical priority is a reproducible classification of the dominant resistance state in an individual tumor. That will require standardized, prospectively validated assessment of antigen presentation, immune-cell location and function, checkpoint activity, and change over time. Spatial and single-cell technologies can support this goal, but their value will be established only if they improve patient selection or treatment decisions beyond conventional clinicopathological variables.

## Figures and Tables

**Table 1:** Components of integrated immune profiling and their potential clinical relevance in cervical cancer.

Immune Parameter	Biological Information Provided	Potential Clinical Relevance	References
**HLA-I expression**	Reflects the capacity of tumor cells to present HPV-derived peptides to CD8^+^ T lymphocytes	Loss or downregulation may indicate impaired tumor recognition and a potential mechanism of resistance to T-cell-mediated immunity; its predictive value for immunotherapy requires prospective validation	[16,17,18]
**CD8^+^ tumor-infiltrating lymphocytes**	Reflect the presence and spatial distribution of cytotoxic immune cells within the tumor microenvironment	Higher infiltration, particularly within tumor nests, may indicate a more active antitumor immune response; prognostic and predictive significance varies according to localization and study methodology	[13,24]
**FOXP3^+^ regulatory T cells**	Indicate the presence of regulatory immune suppression within intratumoral and stromal compartments	Their clinical significance appears context-dependent and may differ according to density, spatial localization, tumor subtype, and accompanying immune-cell populations	[13,26,27,28]
**CD8/FOXP3 ratio**	Represents the relative balance between cytotoxic immune activity and regulatory immune suppression	Biologically informative, but statistically sensitive to sparse cell counts, sampling, compartment definition, and data-derived cut-offs; no clinically validated threshold is available	[24,26,27]
**PD-L1 expression**	Reflects activation of the PD-1/PD-L1 immune checkpoint and adaptive immune resistance	PD-L1 combined positive score is used in specific clinical settings to support selection for pembrolizumab-containing therapy; however, PD-L1 alone does not fully predict treatment response	[11,30,34]
**Spatial immune-cell distribution**	Describes whether immune cells are located within tumor nests or remain restricted to the surrounding stroma	May provide clinically relevant information beyond total immune-cell density and may help distinguish immune-infiltrated from immune-excluded tumors	[13,24,28]
**Integrated immune profile**	Combines antigen-presentation status, immune-cell composition, checkpoint expression, and spatial organization	A research framework rather than a validated clinical assay; high-dimensional models require prespecified features, calibration, external validation, and evidence of added clinical value	[11,13,16,17,18,24,26,27,28,30]

## Data Availability

No new data were created or analyzed in this study. Data sharing is not applicable to this article.

## Abbreviations

CAR-T	chimeric antigen receptor T cell
CCL20	C-C motif chemokine ligand 20
CCR6	C-C chemokine receptor type 6
CD8	cluster of differentiation 8
CD28	cluster of differentiation 28
CD80	cluster of differentiation 80
CD86	cluster of differentiation 86
CD94	cluster of differentiation 94
CIN	cervical intraepithelial neoplasia
CIN2/3	cervical intraepithelial neoplasia grades 2/3
CTLA-4	cytotoxic T-lymphocyte-associated protein 4
DNA	deoxyribonucleic acid
E2	human papillomavirus E2 protein
E2F	E2F transcription factor
E6	human papillomavirus E6 oncoprotein
E7	human papillomavirus E7 oncoprotein
FOXP3	forkhead box P3
HIV	human immunodeficiency virus
HLA	human leukocyte antigen
HLA-I	human leukocyte antigen class I
HPV	human papillomavirus
hrHPV	high-risk human papillomavirus
IFN-γ	interferon gamma
IL-2	interleukin 2
IL-10	interleukin 10
ILT2	immunoglobulin-like transcript 2
LAG-3	lymphocyte-activation gene 3
MDSC	myeloid-derived suppressor cell
MIP-3α	macrophage inflammatory protein 3 alpha
NK	natural killer
NKG2A	natural killer group 2A
PD-1	programmed cell death protein 1
PD-L1	programmed death-ligand 1
RNA	ribonucleic acid
SERPINB3	serpin family B member 3
TAM	tumor-associated macrophage
TAP	transporter associated with antigen processing
TAP1	transporter associated with antigen processing 1
TAP2	transporter associated with antigen processing 2
TCR	T-cell receptor
TGF-β	transforming growth factor beta
TIGIT	T-cell immunoreceptor with immunoglobulin and ITIM domains
TIL	tumor-infiltrating lymphocyte
TIM-3	T-cell immunoglobulin and mucin-domain containing-3
TLR3	Toll-like receptor 3
Treg	regulatory T cell
